# Parental Concerns about the Health of Adolescents with Intellectual Disability: A Brief Report

**DOI:** 10.1155/2011/164080

**Published:** 2011-06-22

**Authors:** Madonna Tucker, Miriam Taylor Gomez, Therese Rey-Conde, Nicholas Lennox

**Affiliations:** Queensland Centre for Intellectual and Developmental Disability, The University of Queensland, Mater Hospital, South Brisbane, QLD, 4101, Australia

## Abstract

*Background*. Parents of adolescents with intellectual disability are concerned about the future health and well-being needs of their children. *Method*. Qualitative data was collected as part of a cross-sectional descriptive study and semi-structured interviews were conducted with 32 parents. The results were themed. *Results*. Most parents discussed areas of their children's health which made them anxious about the future. These concerns were collated into five themes. *Conclusion*. The health and well-being themes were dependency, general health, challenging behaviours, and increasing support needs.

## 1. Introduction

Compared with the general population, adults with intellectual disability experience significant healthcare inequalities including general health screening, mental health support, women's health screening, and oral healthcare services [1], and this equally applies to adolescents with intellectual disability [2, 3]. The majority of Australian adolescents (11–19 years) with intellectual disability live with their parents who have the responsibility for the healthcare of their adolescent child [4, 5]. 

Parents are concerned about the health of their adolescents with intellectual disability [6–9] especially when they can no longer care for them [10]. As adolescents transition out of specialist-based pediatric care, they move to the primary care system and general medical practitioners (GPs); this creates additional parental concerns about their child's health [11, 12]. GPs have expressed concern at being expected to take on a similarly intensive role [13]. This study states the major themes expressed by parents regarding the health of their adolescent with intellectual disability. 

## 2. Method

During a cross-sectional descriptive study which examined the effect of health interventions, interviews with parents were undertaken. This was a six-month cross-sectional descriptive study with qualitative and quantitative data collected. Qualitative data was collected from adolescents with intellectual disability, parents, and the adolescents' teachers. The other findings are reported elsewhere [3]. In a semistructured interview parents were asked about the three main health issues relevant to their adolescent in the next ten years. The dominant themes are discussed here. 

## 3. Results

Thirty-two parents participated, of which 31 were mothers, with a mean age of 46 years, with 21% having tertiary qualifications. They were employed in a range of occupations including home duties, teaching, small business, retail, and nursing. Their adolescent children were described through GP notes and parent/teacher reporting as having mild (3/32), moderate (17/32), severe (11/32), and profound (1/32) intellectual disability. Only 33% (11/32) said that their children were strong and healthy and they had no concerns for the future, and 58% (18/32) said that there were particular areas of their children's health that made them anxious about the future, those being dependency, general health, challenging behaviour, and increasing support needs. 

### 3.1. Theme One: Dependency

Parents made practical observations about the capacity of their children's independence in future health decisions: *“He will always be very dependent on us, and the resources we provide and the professional advice we seek*.*”* A few parents spoke of independence and how the future was not such a worry for them. *“Independence—he likes to have his own things. The (health) diary (the study intervention) is his way of telling people about his health without his mum having to talk all the time*.*”*

### 3.2. Theme Two: General Health

Weight was most frequently named as the biggest challenge. There were additional concerns about management of medication, epilepsy management, *“staying healthy mentally*,*”* mobility reduction over time, *“getting herself to and from the doctor's when she is not feeling well and knowing when to go,” “moving her from the child health to adult health system,”* and maintenance of health checks. 

### 3.3. Theme Three: Challenging Behaviours

Parents consider that others outside the school environment will not know about *“behavioural issues related to his condition.” *Another added that her main concern was that of *“anger management” *and another because *“his behavioural issues may affect his social and daily life*.*”*

### 3.4. Theme Four: Increasing Support Needs

Parents perceived that they will need support for their own health as they age: *“Support structures—we will need support in place as he and I get older”; “Advocacy—someone else needs to be aware of his health needs”; “Someone else to know his normal health-related patterns would be helpful. As his primary care-provider I may not always be around.”*

## 4. Conclusion

These findings are not a comprehensive list of parental concerns, but they contribute to our common understanding of parental health concerns for the future of their adolescent with intellectual disability. 

## References

[1] Emerson E, Durvasula S (2005). Health inequalities and people with intellectual disabilities: an introduction to the special issue. *Applied Research in Intellectual Disabilities*.

[2] Geenen SJ, Powers LE, Sells W (2003). Understanding the role of health care providers during the transition of adolescents with disabilities and special health care needs. *Journal of Adolescent Health*.

[3] Lennox N, Rey Conde T, Faint S (2008). A pilot of healthcare interventions for adolescents with intellectual disability. *Journal of Applied Research in Intellectual Disabilities*.

[4] Australian Institute of Health & Welfare (2003). *Disability Prevalence and Trends*.

[5] Wen X (1997). *The Definition and Prevalence of Intellectual Disability in Australia*.

[6] Abbeduto L, Seltzer MM, Shattuck P, Krauss MW, Orsmond G, Murphy MM (2004). Psychological well-being and coping in mothers of youths with autism. *American Journal on Mental Retardation*.

[7] Browne G, Bramston P (1998). Parental stress in families of young people with an intellectual disability: the nurses role. *The Australian Journal of Advanced Nursing*.

[8] Moos RH (2002). Life stressors, social resources, and coping skills in youth: applications to adolescents with chronic disorders. *Journal of Adolescent Health*.

[9] White N, Hastings RP (2004). Social and professional support for parents of adolescents with severe intellectual disabilities. *Journal of Applied Research in Intellectual Disabilities*.

[10] Henwood M Home and away: carers’ health and well-being in the UK and Australia—similarities and differences.

[11] Fiorentino L, Phillips D, Walker A, Hall D (1998). Leaving paediatrics: the experience of service transition for young disabled people and their family carers. *Health and Social Care in the Community*.

[12] Blum RW, Garell D, Hodgman CH (1993). Transition from child-centered to adult health-care systems for adolescents with chronic conditions: a position paper of the Society for Adolescent Medicine. *Journal of Adolescent Health*.

[13] O’Connell B, Bailey S, Pearee J (2003). Straddling the pathway from paediatrician to mainstream health care: transition issues experienced in disability care. *Australian Journal of Rural Health*.

